# Differential Diagnosis of Multiple Systemic Aneurysms

**DOI:** 10.7759/cureus.30043

**Published:** 2022-10-07

**Authors:** Christian N Schill, Steven Tessier, Santo Longo, Firas Ido, Sudip Nanda

**Affiliations:** 1 Department of Research and Innovation, St. Luke's University Health Network, Bethlehem, USA; 2 Department of Internal Medicine, Lewis Katz School of Medicine, Temple University, Philadelphia, USA; 3 Department of Pathology, St. Luke's University Hospital, Bethlehem, USA; 4 Department of Pulmonary and Critical Care Medicine, St. Luke's University Health Network, Bethlehem, USA; 5 Department of Cardiology, St. Luke's University Hospital, Bethlehem, USA

**Keywords:** pulmonary arterial aneurysms, atypical kawasaki disease, common iliac artery aneurysm, connective tissue disease, vascular ehlers-danlos syndrome, aneurysms-osteoarthritis syndrome, multiple systemic aneurysms

## Abstract

Atherosclerosis and systemic hypertension are the most common pathogeneses of solitary acquired arterial aneurysms. The rare occurrence of multiple synchronous or metachronous arterial aneurysms requires considering alternative underlying causes. We present the unusual case of a male patient who sequentially developed multiple co-existing arterial aneurysms between the ages of 51 and 59. The sites of involvement included high-pressure systemic arteries and low-pressure pulmonary arteries. We discuss the broad differential diagnosis that includes heritable and non-inheritable etiologies. A keen clinical awareness of this broader array of arterial aneurysms is essential for accurate early diagnosis and proper management.

## Introduction

Saccular or fusiform distentions of the arterial wall of the systemic circulation are common findings in the general adult population. Their single most important consequence is catastrophic rupture. The most common location of atherosclerotic aneurysm formation is the abdominal aorta [1]. The development of synchronous and/or sequentially acquired aneurysms, and those involving rare arterial locations, should alert physicians to consider and investigate alternative causes [1]. We present a normotensive patient without evidence of generalized atherosclerosis who developed multiple co-existing acquired systemic and pulmonary arterial aneurysms. This very rare arteriopathy merits a discussion of the differential diagnosis and approaches to clinical management.

## Case presentation

**Figure 1:**
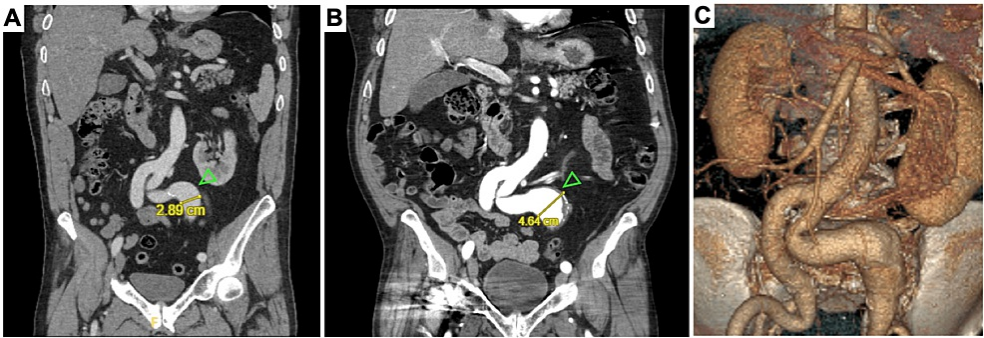
Left iliac artery aneurysm. CT angiograms of the abdomen and pelvis showing the left common iliac artery aneurysm (green arrowheads) on (A) initial presentation, measuring 2.9 cm, and (B, C) 10 years later, measuring 4.6 cm. Also shown is marked tortuosity of the left common iliac artery.

**Figure 2:**
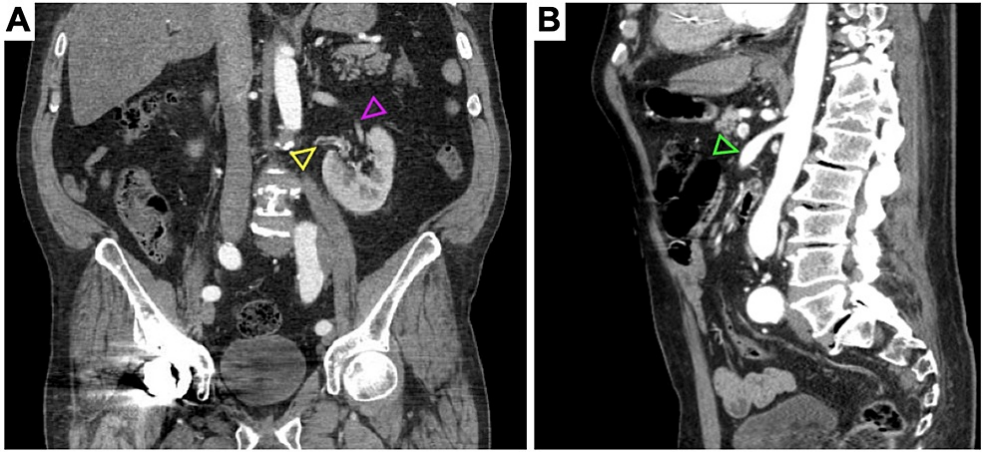
Accessory renal arteries and superior mesenteric artery aneurysm. CT angiograms of the abdomen and pelvis showing (A) the dominant left renal artery (purple arrowhead) arising at the level of the L2 vertebrae, an accessory left renal artery (yellow arrowhead) arising more inferiorly from the descending aorta at the level of L3-L4, and (B) a 1.4-cm aneurysm of the proximal superior mesenteric artery (green arrowhead).

The patient is a 51-year-old man with a one-year history of statin-controlled hyperlipidemia and beta-blocker-controlled atrial fibrillation. He also had a 10-year history of osteoarthritis of the shoulders, knees, and spine with no evidence of joint hyperelasticity or luxation. He was a non-smoker and reported remote social alcohol use. His history did not include significant previous infection or underlying cardiomyopathy. Family history was significant for aneurysms in his mother and father, but the extent and location of their disease are unknown. Family history did not include cardiomyopathy or connective tissue disease. The patient presented to the ED after a 20-foot fall from a ladder onto a concrete surface. CT of the abdomen and pelvis incidentally captured a tortuous and dilated (2.9 cm) common iliac artery (Figure 1). Bilateral duplication of the renal arteries was also incidentally discovered (Figure 2). The patient sustained no serious injuries from his fall and was discharged after his trauma work-up.

**Figure 3:**
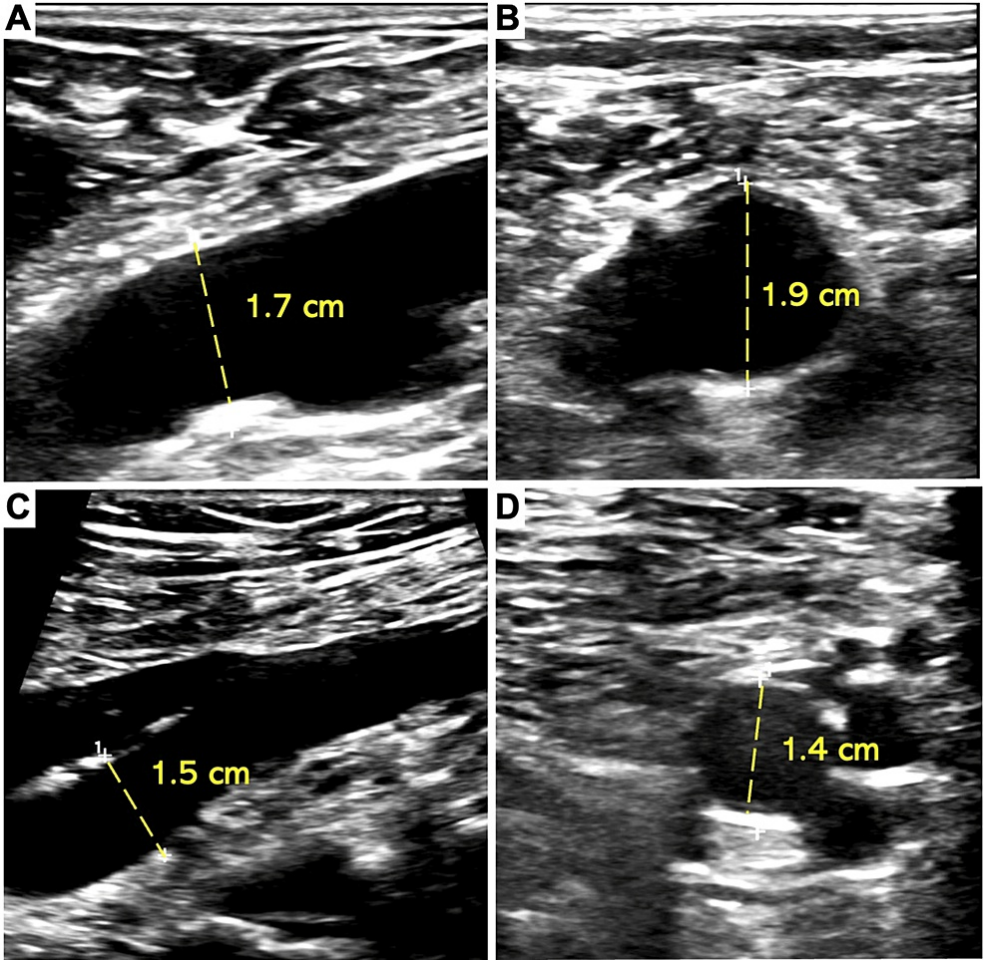
Arterial ultrasound of the bilateral lower limbs. Aneurysms of the (A) right common femoral artery (1.7 cm), (B) left common femoral artery (1.9 cm), (C) right popliteal artery (1.5 cm), and (D) left popliteal artery (1.4 cm).

**Figure 4:**
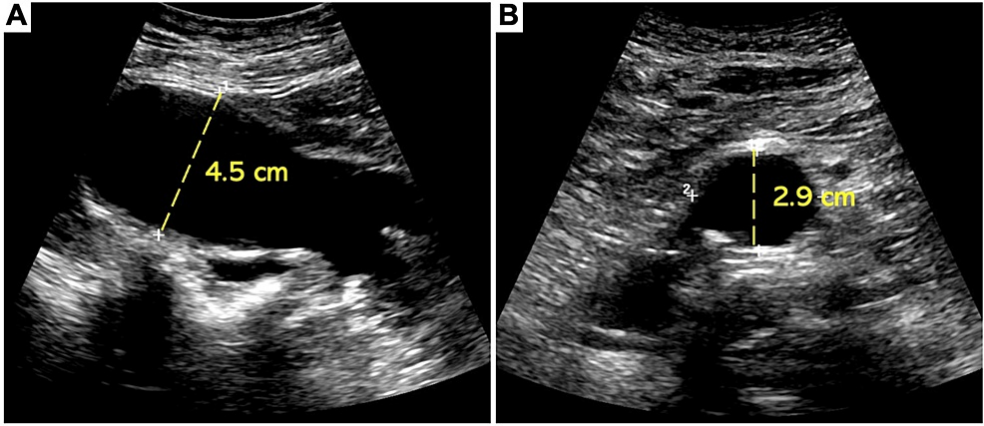
Arterial ultrasound of the abdominal aorta and left common iliac artery. (A) Aneurysm of the left common iliac artery approximately 10 years after its initial discovery, measuring 4.5 cm. (B) Infrarenal abdominal aorta with fusiform dilatation, measuring 2.9 cm.

**Figure 5:**
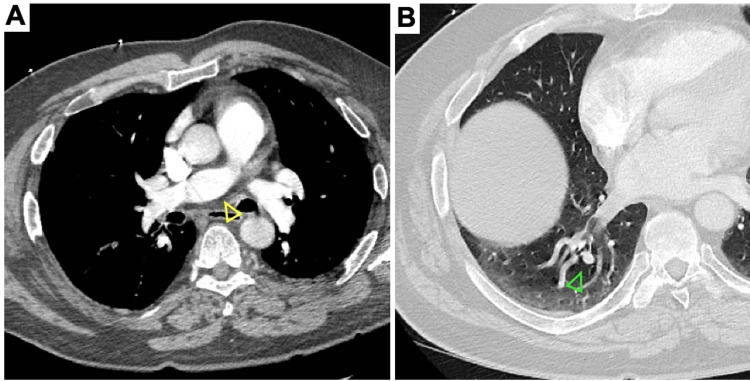
CT angiogram of the chest. (A) Dilatation of the right pulmonary trunk (yellow arrowhead) and (B) intraparenchymal lobar arteriolar ectatic plexus of the right pulmonary vasculature (green arrowhead).

**Figure 6:**
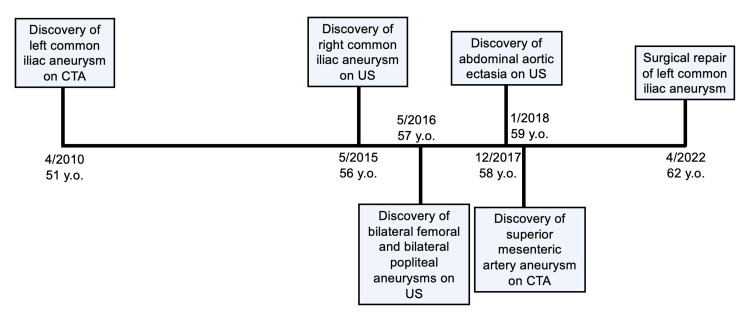
Timeline detailing the patient’s course of aneurysm formation. CTA: Computed tomography angiogram; US: Ultrasound.

Five years later, a new 1.7 cm aneurysmal dilatation of the right common iliac artery was discovered by arterial ultrasound (Figure 3). Additional aneurysms emerged over the subsequent three years. These included 1.7 and 1.9 cm bilateral aneurysms of the femoral arteries, 1.5 and 1.4 cm bilateral aneurysms of the popliteal arteries (Figure 3), ectasia of the infrarenal aorta measuring 2.9 cm (Figure 4), and an aneurysm of the superior mesenteric artery measuring 1.2 cm (Figure 2). Strikingly, heretofore undetected dilatation of the roots of both pulmonary arteries and intraparenchymal lobar arteriolar ectatic plexuses had emerged (Figure 5). A timeline of the patient's course is shown in Figure 6.

All the arterial defects were routinely monitored by arterial duplex ultrasound and CT angiograms. Over the span of 11 years, the left common iliac artery aneurysm enlarged from 2.9 to 4.9 cm and was surgically repaired with an interpositioned arterial graft via retroperitoneal approach (Figure 1). All other aneurysms have remained stable. The patient is currently asymptomatic, and arteriopathy and connective tissue genetic screening are under consideration. In addition, he maintains routine serial follow-up imaging with arterial duplex ultrasounds every six months for surveillance.

## Discussion

True arterial aneurysms present as focal dilatations greater than 1.5 times the normal vessel diameter due to the degeneration of elastin and collagen in the media of the arterial wall [2]. Aneurysms can be classified as saccular or fusiform. Saccular refers to a focal asymmetric outpouching of the arterial wall with the remainder of the vessel circumference unaffected [2]. Fusiform aneurysms are a completely circumferential widening of the vessel wall [2]. Aneurysms involving unusual sites, such as the common iliac, femoral, popliteal, superior mesenteric, and pulmonary arteries, rarely occur [1,2].

Multiple aneurysms in elderly patients are readily ascribed to generalized atherosclerosis. However, the appearance of single or multiple acquired aneurysms in the pre-atherosclerotic age group requires the immediate consideration of possible genetic and/or connective tissue disorders [1]. The broad differential diagnosis of multiple systemic aneurysms includes heritable disorders, non-infectious autoimmune vasculitides, mechanical injuries, and infectious diseases (Table 1).

**Table 1:** Differential diagnosis of multiple systemic aneurysms. Source: Reference [1].

Etiologies	Diagnoses
Genetic Causes	Aneurysms-osteoarthritis syndrome, Ehlers-Danlos syndrome vascular type, Marfan syndrome, Loey’s-Deitz syndrome, Arterial Tortuosity syndrome, Autosomal dominant polycystic kidney disease
Inflammatory	Polyarteritis nodosa, Atypical Kawasaki disease, Behçet's Disease
Infectious Aneurysms	Syphilis, BCG, and Staphylococcus
Degenerative Disease	Athersclerosis

Endovascular and open surgical procedures are the mainstay treatment for aneurysms with a high risk of rupture. Arterial duplex ultrasound can be used for regular monitoring of aortic, iliac, and lower limb aneurysms. CT angiogram is the imaging modality of choice for pulmonary and visceral aneurysms. Consensus has established the aneurysmal diameter at which intervention is mandated. For the aorta in men, the aorta in women, femoral, and popliteal arteries, the diameters that warrant intervention are 5.5, 5.0, 2.5, and 2.0 cm, respectively [1]. Due to rarity, the size threshold for surgical intervention of common iliac aneurysms is not established. However, it is accepted that rupture of common iliac aneurysms measuring less than 4.0 cm is unlikely [3]. As a distinct group, cerebral aneurysms are associated with rupture at 7.0 mm or greater in diameter. The location, morphometry, and individual patient risk factors are important considerations for treatment by endovascular coil embolization or open craniotomy with aneurysmal clipping [4]. Any symptomatic or complicated aneurysm must be surgically treated regardless of size [1]. Medical therapy for all arterial aneurysms strongly relies on accurate diagnosis and etiologic classification (Table 2).

**Table 2:** Treatment modalities by diagnosis. *Currently, there is no consensus for medical management of this disease. Based on its mechanistic similarity to Loey’s-Deitz syndrome, some papers have suggested beta-blockers and ARBs for arterial event prevention [5]. ARBs: Angiotensin receptor blockers.

Diagnosis	Treatment Modalities
Atherosclerosis	Smoking cessation, statin therapy, hypertension control, surgical repair
Aneurysm-Osteoarthritis Syndrome	Arthritic pain control, early elective surgical aneurysm repair*
Ehlers-Danlos Syndrome Vascular Type	Beta-blockers, ARBs, surgical repair
Loey’s-Deitz Syndrome	Beta-blockers, ARBs, surgical repair
Kawasaki Disease	Aspirin, IV immunoglobulins, surgical repair
Polyarteritis Nodosa	Steroids, immune-modifying medications like azathioprine or cyclophosphamide
Infectious Aneurysms	Antibiotics and emergent surgical repair

Atherosclerosis

Among patients with an atherosclerotic abdominal aortic aneurysm, only a small percentage (<4%) also have aneurysms at other sites [6]. A total of 50-90% of patients who have atherosclerotic femoral aneurysms develop a second aneurysm elsewhere [7]. Multiple aneurysms due to diffuse atherosclerosis usually occur in patients over the age of 75 years old [7]. These atherosclerotic aneurysms are typically asymptomatic and found incidentally on imaging [2]. Risk factors include smoking, male sex, hypertension, and hyperlipidemia [1]. Treatment involves smoking cessation, blood pressure control, statin therapy, and surgical repair [1]. Our patient presented with an expanding left common iliac aneurysm at the relatively young age of 51 years old. His hyperlipidemia and hypertension have been well-controlled with medical therapy, his alcohol use history is remote, and his smoking history was minimal. The coronary narrowing was insignificant upon review by recent myocardial perfusion imaging. An underlying genetic condition was strongly considered to be the driving cause of our patient's multiple aneurysms by age and anatomic location.

Genetic etiologies

The recently described aneurysm-osteoarthritis syndrome (AOS) is a plausible cause of our patient's presentation [8,9]. AOS is characterized by multiple systemic aneurysms and early-onset osteoarthritis [8,9]. This syndrome is caused by autosomal dominant mutations in *SMAD3* [9]. S*MAD3* is a signaling protein of the transforming growth factor $\beta$ (TGF-$\beta$) pathway [9]. In AOS, *SMAD3* mutations lead to its own overexpression and, consequentially, the overexpression of multiple genes also involved in the TGF-$\beta$ signaling pathway [9]. Excessive TGF-$\beta$ signaling is associated with aneurysm formation and blood vessel tortuosity [9]. The aneurysms of AOS typically affect the common iliac artery, aorta, and/or visceral arteries. Arterial tortuosity has also been described. Our patient manifested each of these components. Other findings associated with AOS include a long face, hypertelorism, flat orbital ridges, malar flattening, bifid uvula, aortic dissection, and cutaneous telangiectasias [9]. However, 50-70% of patients with AOS do not have craniofacial dysmorphism [9]. While pulmonary artery involvement has yet to be described for AOS, it has been described in similar genetic disorders [10]. Although no clinical management is clearly established, the treatment of aneurysms relies upon faithful routine surveillance [8]. Multiple commercially available genetic screens for connective tissue diseases include *SMAD3* to confirm the diagnosis.

Ehlers-Danlos syndrome (EDS) is caused by genetic defects in collagen [11]. EDS has multiple subtypes with various phenotypic presentations [11]. EDS type IV, also called vascular type EDS, is associated with mutations in the *COL3A1 *gene, which encodes type III collagen of arterial walls and hollow organs [12]. This EDS type is associated with arterial fragility and the formation of aneurysms in the central and peripheral nervous systems. The average life expectancy of patients with vascular EDS is 48 years old due to the complications of friable intracranial aneurysms, dissection of cervical vessels, carotid-cavernous fistulas, and uterine or bowel rupture. Wide-spread systemic arterial aneurysms may occur in EDS type IV. Marfan's syndrome is an autosomal dominant disorder associated with mutations in the *FBN1* gene, which encodes fibrillin-1 [13]. Characteristic features of Marfan's syndrome include pectus excavatum, ectopia lentis, ascending aortic dilatation, aortic dissection, mitral valve prolapse, and arachnodactyly [13]. Aneurysms typically occur in the aorta and are rarely found in the systemic arteries [13]. While rare, pulmonary artery aneurysms have also been described in patients with both Marfan's and Ehler-Danlos's diseases [10].

Tortuous vessel arteriopathies are associated with multiple aneurysm disease [14]. The arteriopathy most associated with tortuosity and the formation of numerous aneurysms is Loey's-Deitz syndrome [14]. Loey's-Deitz syndrome is an autosomal dominant disorder caused by gene mutations that encode components of the TGF-$\beta$ signaling pathway, typically *TGFBR1* or *TGFBR2* [14]. Loey's-Deitz syndrome presents with congenital craniofacial malformations, arterial tortuosity, and systemic aneurysms [14]. The aortic root and arteries of the head and neck are characteristically affected [14]. Management of Loey's-Deitz syndrome includes surveillance for the development of aneurysms [14]. Starting in childhood, therapy with beta-blockers and losartan may slow aortic root dilation but not the need for aortic repair later in life [14]. Arterial tortuosity syndrome may also be considered in patients with significant multi-vessel tortuosity in addition to aneurysms, but the tortuosity in this patient was confined to a single artery.

Autosomal dominant polycystic kidney disease (ADPKD) is associated with mutations in either PKD1 or PKD2 [15]. ADPKD classically presents with hypertension, excessive bilateral kidney cysts, liver cysts, and an increased risk of intracranial berry aneurysms [15]. In addition, a few case reports have documented the presence of extracranial aneurysms in the common iliac, celiac, gastric, and splenic arteries [15].

Non-infectious inflammation

Inflammatory vascular disorders of medium-sized vessels, such as atypical (incomplete) Kawasaki disease, polyarteritis nodosa, and Behçet's disease, should be considered in patients presenting with multiple systemic aneurysms [2,16]. In our patient, the presence of pulmonary aneurysms warrants special consideration of an underlying vasculitis, as inflammatory vessel disease is more likely to involve the pulmonary tree than degenerative or genetic causes [10].

Typically affecting the pediatric patient, Kawasaki disease is a vasculitis that may present with flu-like prodrome, conjunctivitis, strawberry tongue, cutaneous manifestations, and aneurysms of the coronary arteries [16]. The diagnosis is called atypical or incomplete Kawasaki disease when presenting in the adult patient or without meeting all diagnostic criteria [16]. The disease is self-limited and typically lasts 5-12 days if left untreated [16]. Approximately 2% of untreated patients will develop medium-sized artery aneurysms, which most commonly affect the coronary arteries in the weeks to years that follow diagnosis [16]. In one case report, the pulmonary arteries were also affected [16]. Treatment includes aspirin, steroids, and IV IgG to reduce inflammation and prevent further aneurysm formation [16]. Our patient had no aneurysms of the coronary arteries or clinical signs of Kawasaki disease. However, Kawasaki disease must be considered if the cardiac complications associated with this disease are to be prevented by early intervention. Characteristically seen in middle-aged patients, polyarteritis nodosa may cause microaneurysms of the hepatic, renal, and mesenteric arteries [17]. These microaneurysms are often associated with adjacent regions of vascular constriction, which results in ischemic damage to multiple organs. Other associated symptoms include fever, weight loss, abdominal pain, nausea and vomiting, renal infarcts, hematuria, proteinuria, and neuropathy [17]. Treatment for polyarteritis nodosa includes steroids and a second immunosuppressing agent, such as cyclophosphamide [17]. Behçet's disease can present with multiple systemic aneurysms, including the arteries of the pulmonary tree, and characteristically presents with cutaneous and mucosal ulcers of the genitals, eyes, and/or mouth [10,18]. Treatment consists of steroids and immunosuppressive drugs; however, endovascular intervention may be necessary if the patient has pulmonary symptoms [18]. Our patient presented without ulcers, renal signs, GI symptoms, or neuropathy, making neither diagnosis likely.

Infectious causes

Infectious organisms associated with multiple mycotic aneurysms include *T. pallidum*, *S. pneumoniae*, *S. enterocolitica*, and* M. Bovis* (BCG) [1,19,20]. Live attenuated BCG organisms are used in the treatment of urothelial cancer. Albeit rare, these organisms may cause bacteremia and consequential mycotic aneurysm formation [20]. The destructive bacterial invasion of the arterial wall can lead to rapidly progressive aneurysmal dilatation in a matter of days or weeks [19]. Mycotic aneurysms are more prone to rupture than non-infectious etiologies [1]. Any vessel may be infected; however, the most prominently are the aorta and coronary, femoral, and popliteal arteries [19,20]. Of note, syphilis and tuberculosis have been reported in rare instances to cause pulmonary aneurysms [10]. Symptoms of mycotic aneurysms may include fever, malaise, pain, and point tenderness at the site of the aneurysm [19, 20]. Treatment regimens are determined on a case-by-case basis [19, 20].

## Conclusions

Physician awareness of multiple acquired arterial aneurysms affecting common and/or atypical arterial sites, particularly in the younger patient, requires knowledge and recognition of rare non-atherosclerotic arteriopathies. The temporal nature of aneurysm formation and its associated symptoms provide clues for potential diagnosis. While infectious aneurysms present over weeks, inflammatory aneurysms usually present over weeks to months. Early detection and treatment of inflammatory aneurysms are paramount for preventing further damage to the vasculature. Infectious aneurysms are particularly dangerous and should always be excluded. Pulmonary aneurysms are more likely to result from inflammatory and infectious causes than genetic diseases. Slow development of aneurysms over many years without systemic signs of infection or inflammation should prompt consideration of inherited connective tissue disorders or tortuous arteriopathies. Genetic panels for such diagnoses are commercially available. Confirmation of a genetic cause may also inform the care of the patient's family members. The inheritance of AOS, Marfan's syndrome, Vascular Ehlers-Danlos syndrome, and Loey's-Deitz syndrome is autosomal dominant; if any of these diseases are identified in a patient, his/her children must be genetically screened. Management of multiple aneurysms involves prophylactic medical therapy and routine monitoring of aneurysm size.
